# Interactive Relationships between Intestinal Flora and Bile Acids

**DOI:** 10.3390/ijms23158343

**Published:** 2022-07-28

**Authors:** Xiaohua Guo, Edozie Samuel Okpara, Wanting Hu, Chuyun Yan, Yu Wang, Qionglin Liang, John Y. L. Chiang, Shuxin Han

**Affiliations:** 1 Department of Hepatobiliary Surgery, Anhui Province Key Laboratory of Hepatopancreatobiliary Surgery, The First Affiliated Hospital of USTC, Division of Life Sciences and Medicine, University of Science and Technology of China, Hefei 230001, China; xiaohuaguo@mail.ustc.edu.cn (X.G.); edosam2032@gmail.com (E.S.O.); ycychuyun@163.com (C.Y.); 2 MOE Key Laboratory of Bioorganic Phosphorus Chemistry & Chemical Biology, Beijing Key Lab of Microanalytical Methods & Instrumentation, Center for Synthetic and Systems Biology, Department of Chemistry, Tsinghua University, Beijing 100084, China; hwt0723@163.com (W.H.); stonewang04@163.com (Y.W.); liangql@tsinghua.edu.cn (Q.L.); 3 Department of Integrative Medical Sciences, Northeast Ohio Medical University, Rootstown, OH 44272, USA

**Keywords:** bile acids, intestinal flora, homeostatic imbalances, diseases, interactions

## Abstract

The digestive tract is replete with complex and diverse microbial communities that are important for the regulation of multiple pathophysiological processes in humans and animals, particularly those involved in the maintenance of intestinal homeostasis, immunity, inflammation, and tumorigenesis. The diversity of bile acids is a result of the joint efforts of host and intestinal microflora. There is a bidirectional relationship between the microbial community of the intestinal tract and bile acids in that, while the microbial flora tightly modulates the metabolism and synthesis of bile acids, the bile acid pool and composition affect the diversity and the homeostasis of the intestinal flora. Homeostatic imbalances of bile acid and intestinal flora systems may lead to the development of a variety of diseases, such as inflammatory bowel disease (IBD), colorectal cancer (CRC), hepatocellular carcinoma (HCC), type 2 diabetes (T2DM), and polycystic ovary syndrome (PCOS). The interactions between bile acids and intestinal flora may be (in)directly involved in the pathogenesis of these diseases.

## 1. Introduction

Intestinal microorganisms are composed of bacteria, archaea, eukaryotes, and viruses, and more than 99% of them are bacteria [1]. Approximately 10^14^ bacteria are known to constitute the intestinal flora in the adult gut, and this number is 10 times the number of human somatic cells [2]. The intestinal flora co-exists harmoniously with the host, participate in the digestion and the absorption of nutrients, and also help to maintain the integrity of the host’s immune system so as to prevent pathogen colonization [3]. Additionally, intestinal flora consists of various bacteria in low or high abundance, which co-evolve with the host. While the host provides nutrients and a suitable survival place for the intestinal flora, the intestinal flora assists the host in absorbing nutrients, such as vitamins and short-chain fatty acids, in a more efficient manner in order to drive growth processes and to support the functions of the intestinal system and the immune system [4].

Bile acids are a group of hydroxylated steroid acids, consisting of a 24-carbon steroid nucleus and a 5-carbon side chain with a carboxyl group at the C-24 position. As the main components of bile, bile acids, in addition to possessing anti-bacterial activity, are amphipathic molecules with physiological detergent features. They are indispensable for emulsifying and absorbing lipid-soluble nutrients and drugs [5,6,7]. Bile acids also function as signaling molecules to bind and to activate a membrane G-protein-coupled bile acid receptor 1 (GPBAR1 or Takeda G-protein-coupled receptor 5 (TGR5)) and a nuclear receptor—the farnesoid X receptor (FXR)—involved in the regulation of lipid, glucose, and energy metabolism [8]. Approximately 80 known bile acids have been identified in mammals, and these include bile acids synthesized in the liver and secondary bile acids produced based on modifications caused by intestinal bacteria [9].

Gastrointestinal homeostasis is subject to the joint regulation of bile acids and intestinal bacteria. Many types of bacteria reside in the intestine, and they are involved in the regulation of the synthesis, metabolism, and reabsorption of bile acids. In turn, bile acid pool and composition, regulate the growth and the population of bacteria in the intestine. As bile acids are continuously circulated within the enterohepatic circle, they undoubtedly play a critical regulatory role in the gut—liver and microbial—host axes. Interestingly, intestinal flora participates in the transformation of bile acids into more hydrophobic secondary bile acids, which modulate immune responses to pathogens [10]. In this review, we summarize the distribution of intestinal flora in different age groups and gastrointestinal fragments, and the physiological characteristics of bile acids. Additionally, we classify the microbial communities in the digestive tract on the species, features, and nature of their relationships with the host. We conclude that while the intestinal flora modulates the metabolism, synthesis, and reabsorption of bile acids, bile acids control the growth and diversity of the intestinal flora. Homeostatic imbalances in this crucial bidirectional interaction may lead to the development of pathologies such as inflammatory bowel disease (IBD), hepatocellular carcinoma (HCC), colorectal cancer (CRC), type 2 diabetes (T2DM), and polycystic ovary syndrome (PCOS).

## 2. Architecture and Composition of the Intestinal Flora

The gut microbiota exists throughout the life of the host. The diversity of bacteria in the intestines of infants is very low at first, and it gradually accelerates during the course of early development. The intestinal floras in newborn babes are mainly of the *Enterobacteriaceae* and *Staphylococcus* species, and the intestinal flora during lactation are mainly of the *Bifidobacterium* species. After the consumption of a solid diet, the bacteria colonizing the intestine are found to be mostly the anaerobic strains [11,12,13]. A low level of *Bacteroidetes* and a high level of *Bifidobacterium* are also found in adolescence, followed by the formation of intestinal microbial communities dominated by Bacteroidetes and Firmicutes, which are involved in carbohydrate and amino acid metabolism, fermentation, and oxidative phosphorylation [14,15]. Studies have shown that aging is associated with a number of important changes, including a decrease in the diversity of the intestinal flora; decreases in the proportions of Firmicutes and Bacteroidetes; decreases in the abundances of *Ruminococcaceae*, *Lachnospiraceae*, and *Bacteroidaceae*; increases in the abundances of opportunistic pathogens; and decreases in the populations of the bacteria crucial for producing short-chain fatty acids required for the maintenance of structural integrity and the prevention of inflammation in the intestine [16,17,18].

**Figure 1:**
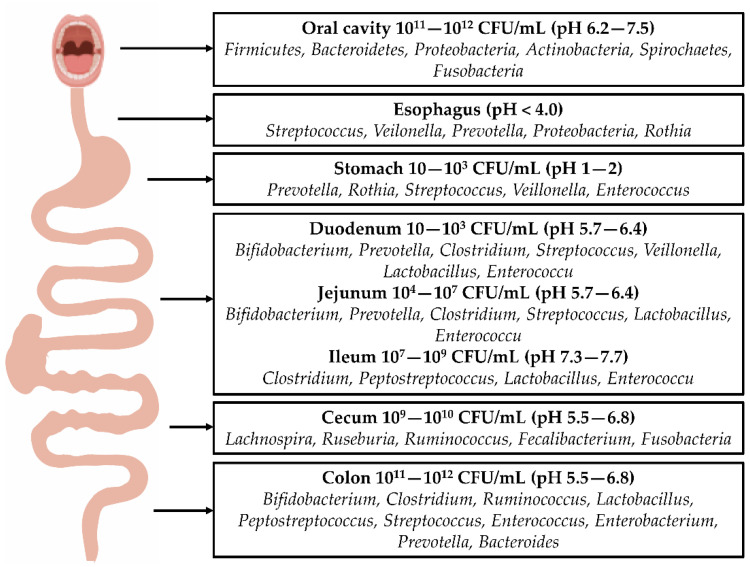
Distribution of gastrointestinal bacteria: The distribution of intestinal bacteria in the digestive tract varies, and there are many types and quantities of bacteria in the oral cavity. Following their entry into the esophagus, the colonization of bacteria is reduced. Due to the secretion of gastric acid, most bacteria in the stomach cannot survive, allowing more acid-tolerant bacteria, such as *Prevotella*, *Roche*, and *Streptococcus*, to dominate. The number of bacteria increases from the duodenum to jejunum and ileum. These bacteria include *Clostridium*, *Lactobacillus*, and *Enterococcus*. A large number of bacteria exist in the colon, including *Bifidobacterium*, *Clostridium*, *Ruminococcus*, *Bacteroides*, *Streptococcus*, and *Prevotella*.

The composition of the intestinal microbiota varies throughout the digestive tract. Food is mixed with saliva before entering the stomach and intestine. The oral microbiota is complex and diverse: ~1000 species of bacteria have been identified to date [19,20]. Esophageal microbial communities mainly include Firmicutes, Bacteroidetes, Actinobacteria, Proteobacteria, and Fusobacteria [21,22]. Most of the bacteria in the host’s body are localized in the gastrointestinal tract, and there are significant differences in bacterial diversity and quantity between the stomach and the intestine. There are 10 to 10^3^ bacteria per gram of stomach content, mainly including Firmicutes, Bacteroidetes, *Clostridium*, Actinobacteria, along with *Streptococcus* and *Haemophilus*. *Helicobacter pylori* is the dominant bacterium in the stomach [23,24]. The small intestine consists of the duodenum, jejunum, and ileum. There are 10^3^ bacteria per gram of duodenal content, and Firmicutes and Actinobacteria are the main bacteria [16]. The bacterial density in the jejunum is high; there are 10^4^–10^7^ bacteria per gram of content—mainly Gram-positive aerobic bacteria and facultative anaerobic bacteria, such as *Lactobacillus*, *Enterococcus*, and *Streptococcus*. The numbers of ileal anaerobic bacteria close to the ileocecal valve, gradually exceed those of aerobic bacteria, and *Streptococcus* is the dominant bacteria in this segment of the intestine [25]. The colon, located in the lower part of the large intestine, contains 10^11^–10^12^ bacteria per gram of content, which are mainly anaerobic bacteria, including Firmicutes and Bacteroidetes. There is a high population density and diversity. The ratio of Firmicutes to Bacteroidetes is related to the susceptibility to diseases. In the large intestine, *Bacteroides*, *Bifidobacterium*, *Streptococcus*, *Enterobacteriaceae*, *Enterococcus*, *Clostridium*, *Lactobacillus*, and *Ruminococcus* are the dominant bacteria. In addition, the colon also contains several pathogenic bacteria, such as *Campylobacter jejuni*, *Salmonella enteritidis*, *Vibrio cholerae*, *Escherichia coli*, and *Bacteroides fragilis* [23]. The distribution of bacteria in the digestive tract is shown in Figure 1.

The intestinal flora is mainly classified according to natural attributes, including Firmicutes, Bacteroidetes, Proteobacteria, Actinobacteria, Verrucomicrobia, Fusobacteria, and Cyanobacteria. Approximately 98% of the intestinal flora are composed of four main types of bacteria—Firmicutes, Bacteroidetes, Proteobacteria, and Actinomycetes—and the classification of the bacteria is shown below in Table 1. The most common bacterial genera are *Bacteroides*, *Clostridium*, *Peptococcus*, *Bifidobacterium*, *Eubacterium*, *Ruminococcus*, *Enterococcus faecalis*, and *Peptostreptococcus* [17]. Furthermore, most of the bacteria in Bacteroidetes belong to *Bacteroidetes* and *Prevotella*, and the Firmicutes are mainly *Clostridium*, *Eubacteria*, and *Ruminococcus*.

In addition to the classification according to natural properties, intestinal flora can be classified according to their relationships with the host. The relationship with the host can be mutually beneficial (i.e., symbiotic), conditionally pathogenic, or exclusively pathogenic. The beneficial bacteria mainly promote intestinal peristalsis, prevent constipation and diarrhea, promote the synthesis of vitamins, discharge exogenous harmful substances, and occlude the invasion of pathogens, including *Bifidobacterium*, *Lactobacillus*, *Lactococcus*, *Enterococcus faecalis*, *Eubacterium*, *Peptococcus*, *Clostridium*, and *Rothia* [26,27]. Under certain conditions, conditionally pathogenic bacteria are invasive and cause harm to the human body. The conditionally pathogenic microorganisms often include *Escherichia coli*, *Enterococcus*, *Ruminococcus*, *Bacteroides*, *Vibrio desulphurization*, *Candida albicans*, and *Pseudomonas aeruginosa* as well as Proteobacteria [28]. Pathogenic bacteria generate toxic metabolites, which increase the reabsorption of harmful substances in the intestine, thereby causing abnormalities in intestinal peristalsis and heightened invasion of the intestinal tract by pathogenic bacteria, including *Escherichia coli*, *Staphylococcus*, Proteobacteria, *Streptococcus*, *Peptostreptococcus*, Fusobacteria, *Clostridium*, *Klebsiella*, *Prevotella*, *Clostridium tetanus*, and *Veillonellaceae* [29,30].

## 3. Bile Acids

Bile acids are steroid molecules synthesized from the precursor cholesterol in the liver by a series of 17 enzymes. Two synthetic pathways have been identified for the synthesis of bile acids: the classical pathway and the alternative pathway [31]. The classical pathway is initiated by the rate-limiting enzyme cholesterol 7α-hydroxylase (CYP7A1) together with a series of hydroxylation, isomerization, and steroid side-chain oxidation and cleavage reactions—including the sterol 12α-hydroxylase and the steroid 27-hydroxylase—to synthesize two primary bile acids: cholic acid (CA) and chenodeoxycholic acid (CDCA). The alternative pathway is initiated by the steroid 27-hydroxylase and the oxysterol 7α-hydroxylase to mainly synthesize CDCA [32]. Bile acids are conjugated with glycine and taurine by bile acid coenzyme A synthase and bile acid: amino-acid transferase at the C24 position in the liver to form conjugated bile acids and to increase their solubility [33,34]. The conjugated bile acids in the liver are excreted by the bile salt export pump (BSEP), and the sulfated and glucuronidated bile acids produced by sulfotransferase and UDP-glucuronosyltransferase are transported by multidrug resistance protein 2 [35]. Importantly, bile acids are temporarily stored in the gallbladder and secreted into the small intestine after food is ingested. Secondary bile acids are produced under the action of intestinal bacteria, and approximately 95% of bile acids are actively reabsorbed in the ileum and terminal colon by the apical sodium/bile acid transporter (ASBT) [9]. Inside intestinal cells, bile acid binds to ileal bile acid-binding protein (IBABP), allowing for its secretion to the portal vein by basolateral organic solute transporter α/β (OST-α/OST-β) and finally uptake into liver cells by sodium taurocholate co-transporting polypeptide (NTCP) and organic anion transporting polypeptide 1 (OATP1) [36,37]. In the liver, free bile acids are re-conjugated with glycine/taurine, and they re-enter the intestine with bile composed of reabsorbed and newly synthesized conjugated bile acids. The repeating circulation process of bile acids between the liver and the intestine is known as enterohepatic circulation [38,39]. Notably, only ~5% of bile acids are disposed via feces, and the portion of bile acids lost in feces is replenished by the synthesis in the liver. The human body experiences approximately 8–10 cycles of enterohepatic circulation daily, which is critical for the regulation of lipid metabolism and homeostasis [40,41].

Notably, great differences exist in the composition of bile acids between humans and mice, which is caused by the species–specific transformation of many bile acids in humans and mice. Bile acids in the human body are mainly composed of CA, CDCA, and deoxycholic acid (DCA) at a ratio of around 40:40:20. The ratio of glycine to taurine conjugated bile acids is approximately 3:1, and together those conjugated bile acids account for more than 90% of human bile acids [42]. DCA is synthesized from CA under the action of intestinal bacterial enzymes but differs from CA in the number of hydroxyl groups. The amounts of lithocholic acid (LCA) and ursodeoxycholic acid (UDCA) circulating in the human body are low, at approximately 2–5%. UDCA is a secondary bile acid produced by intestinal-bacteria-mediated 7-OH isomerization of CDCA [9]. The bile acids in the gallbladders of healthy individuals are mainly composed of CA, CDCA, and DCA. DCA and LCA are the main bile acids in feces. The differences in bile acid components in the gallbladder and the feces reflect the degree of bile acid modification by intestinal bacteria [43]. In mice, most bile acids are taurocholic acid (TCA), tauro-β-muricholic acid (T-βMCA), T-αMCA, and T-ωMCA [44]. Moreover, UDCA is a primary bile acid generated by the enzymatic action of CYP2C70 in mice. CDCA and UDCA undergo 6β-hydroxylation to produce αMCA and βMCA—the more hydrophilic bile acids that are abundant in mice [45,46]. LCA can be converted to UDCA by 7α-hydroxylase, to hyodeoxycholic acid (HDCA) by 6α-hydroxylase, or to murideoxycholic acid (MDCA) by 6β-hydroxylase [47]. The gut bacteria also synthesize very small amounts of oxo-, iso-, and epi-bile acids. The diversity of secondary bile acids and the composition of bile acid pools are influenced by intestinal microbes in different individuals. The gut microbiome can regulate the production of secondary bile acids, which in turn affects bile-acid-mediated digestive, absorptive, signaling, and other functions. The structures of bile acids are shown in Table 2.

On the basis of whether the C12 position contains a hydroxyl (-OH) group, bile acids can be divided into 12-OH and non-12-OH bile acids. This enzymatic process is mainly catalyzed by CYP8B1, which can determine the ratio of non-12-OH to 12-OH bile acids in addition to the bile hydrophobicity by promoting the biosynthesis of CA [48]. The 12-OH bile acids mainly include CA, DCA, UCA, and T/G-conjugated bile acids. Non-12-OH bile acids mainly include CDCA, LCA, MCA, MDCA, UDCA, HCA, HDCA, and T/G-conjugated bile acids. Notably, an increased ratio of 12-OH to non-12-OH bile acids is mostly associated with metabolic diseases, such as obesity, diabetes, and nonalcoholic fatty liver disease [49]. Studies have shown that TDCA or GDCA can promote the proliferation of hepatic stellate cells and the formation of hepatic fibrosis by activating the TGR5-ERK1/2 and P38 MAPK signaling pathways [50].

Finally, according to the number of hydroxyl groups, bile acids can also be divided into mono-, di- and tri-hydroxy bile acids. The hydrophilicity of bile acids is proportional to the number of hydroxyl groups they contain, and it also depends on the position and the stereochemical structure of the hydroxyl groups. The formation of 7β-OH, 6α-OH and 6β-OH leads to more hydrophilic bile acids than the modification with 7α-OH. UDCA containing 7β-OH is the most hydrophilic bile acid among the whole group of di-hydroxy bile acids. Furthermore, MCA (6α-OH, 6β-OH) and HCA (6α-OH) are more hydrophilic tri-hydroxy bile acids than other tri-hydroxy bile acid subfamily members.

## 4. Interaction between Intestinal Flora and Bile Acids

The gastrointestinal microbiota is a natural ecosystem crowded with more than 10^14^ bacteria. Approximately 99% of the functional genes in the human body are derived from microorganisms, and these genes play multiple regulatory roles—particularly the production of secondary bile acids by the intestinal flora to regulate the pathophysiological functions of the host [51]. Secondary bile acids are produced from host-synthesized primary bile acids by the intestinal flora. Therefore, intestinal flora diversifies the bile acids of the host and also, in combination with the primary bile acid metabolism, directly affects the composition, size, and concentration of the bile acid pool in the host [52]. The bile acid pool is a synergistic readout between host and intestinal flora. The diversity of bile acids affects the growth and the proliferation of the intestinal flora to shape the microbial community in the intestine. In the gut microbiome–bile acid–host axis, bile acids can regulate the gut microbiome, and vice versa the gut microbiome regulates the bile acid pool, and those mutual influences are heavily involved in the regulation of multiple diseases such as IBD, CRC, HCC, T2DM, and PCOS.

### 4.1. Effects of the Intestinal Flora on Bile Acids

#### 4.1.1. The Intestinal Flora Is Involved in Bile Acid Metabolism

The effects of the intestinal flora on bile acid composition and secondary bile acid synthesis mainly include four aspects: deconjugation, dehydroxylation, and oxidation isomerization of bile acids.

- Deconjugation

**Figure 2:**
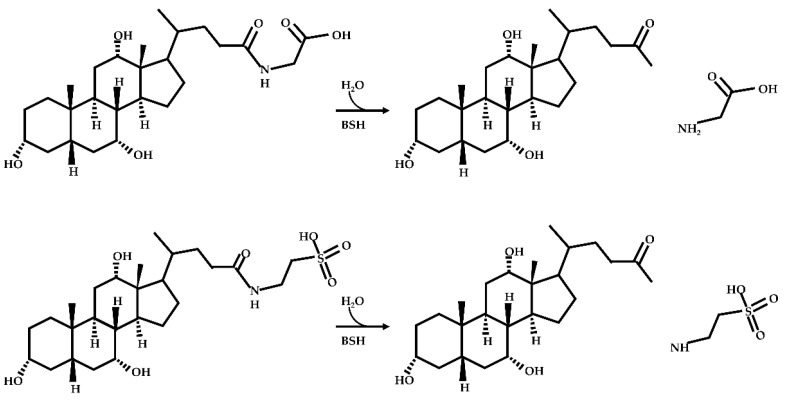
Deconjugation pathways of bile acids: Hydrolysis of conjugated bile acids to free bile acids and glycine or taurine by bile salt hydrolase (BSH).

Deconjugation of bile salts is a pervasive function of the gut microbiota, and is catalyzed by bile salt hydrolase (BSH) enzymes to generate free bile acids with a C-24 carboxylic acid group. BSH is an enzyme that catalyzes the hydrolysis of the amide bonds between the C-24 position of the bile acids and the acidic side of amino acids. BSH belongs to the cholylglycine hydrolase family, which contains at least nine members. The subunit size and composition, optimal pH value, kinetic characteristics, substrate specificity, and regulation of all BSH family members are different [43,53,54]. BSH is widely expressed in the intestinal flora and enriched in the ileum and colon. Mostly, BSH containing bacteria are Gram-positive bacteria, such as *Bifidobacterium*, *Lactobacillus*, *Clostridium*, *Enterococcus*, and *Listeria*, along with Gram-negative bacteria, such as *Bacteroides*, *Stenotrophomonas*, and *Brucella* [55,56]. There are three main hypotheses for BSH function: First, BSH converts conjugated bile acids to free bile acids, allowing for the release of glycine or taurine, both of which can serve as an energy resource—glycine is catabolized to ammonia and carbon dioxide, and taurine is catabolized to ammonia, carbon dioxide, and sulfate [57,58,59]. Second, BSH is thought to integrate cholesterol into bacterial membranes through the formation of intermolecular bonds between bile salts and membrane fatty acids, which may change the fluidity and charge of the membranes [60]. Finally, BSH is also known to play a role in Gram-positive bacteria tolerance by generating free bile acids as a detoxification mechanism [61]. The deconjugation pathways of bile acids are shown in Figure 2 below.

- Dehydroxylation

**Figure 3:**
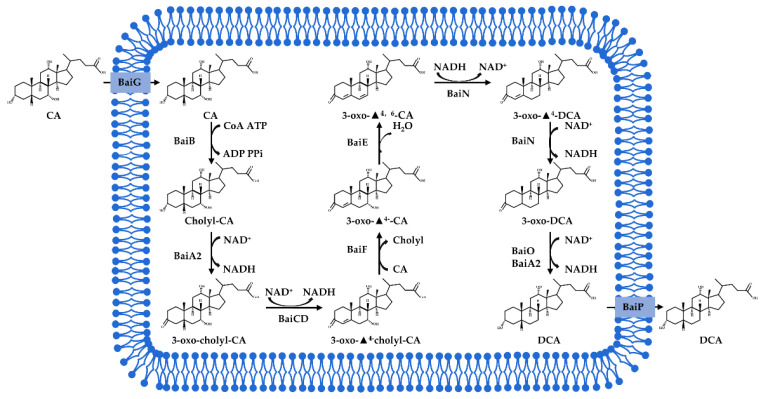
Biotransformation of primary bile acids by *Clostridium species*: Primary bile acids (CA and CDCA) are biotransformed to the secondary bile acids DCA and LCA, respectively. The processes include multiple steps catalyzed by several enzymes and bile acid transporters encoded by bile acid-inducible (BAI) genes.

Bile acid dehydroxylation is one of the key transformations mediated by intestinal microorganisms. Bacterial 7α/β-dehydroxylase activities in the anaerobic, Gram-positive phylum Firmicutes (genera *Clostridium*, *Enterococcus*, *Bifidobacterium*, *Listeria*, and *Lactobacillus*) and Gram-negative phylum Bacteroidetes (genus *Bacteroides*) convert the primary bile acids, CA, and CDCA to the secondary bile acids, DCA and LCA, respectively. The polycistronic operons of these bacteria consist of bile-acid-inducible (Bai) genes in several species of *Clostridium* [43,44]. For example, CA is transported into bacteria through BaiG; subsequently, BaiB links CoA in an ATP-dependent manner to form cholyl-CoA. Cholyl-CoA is first oxidized by BaiA2 and then twice by BaiCD to generate 3-oxo-Δ4-cholyl-CoA. BaiF then transfers CoA from 3-oxo-Δ4-cholyl-CoA to CA, generating 3-oxo-Δ4-CA and cholyl-CoA. 3-Oxo-Δ4-CA is then dehydroxylated at the position of C7 by BaiE, which is followed by the step of deoxycholic acid reduction by BaiN and BaiA2 [62,63,64,65]. Importantly, BaiA, BaiB, and BaiE are three key enzymes in the 7α -dehydroxylation pathway of bile acids. The BaiB gene encodes bile acid coenzyme A ligase, which catalyzes the thioesterification of bile acids to CoA after the primary bile acids are taken up by bacterial cells [66,67]. BaiA encodes 3α-HSDH, and the BaiE gene encodes bile acid 7α-dehydratase catalyzing the rate limiting and irreversible step in the bile acid 7α-dehydroxylation pathway. The dehydroxylation pathways of bile acids are shown below in Figure 3.

- Oxidation and isomerization

**Figure 4:**
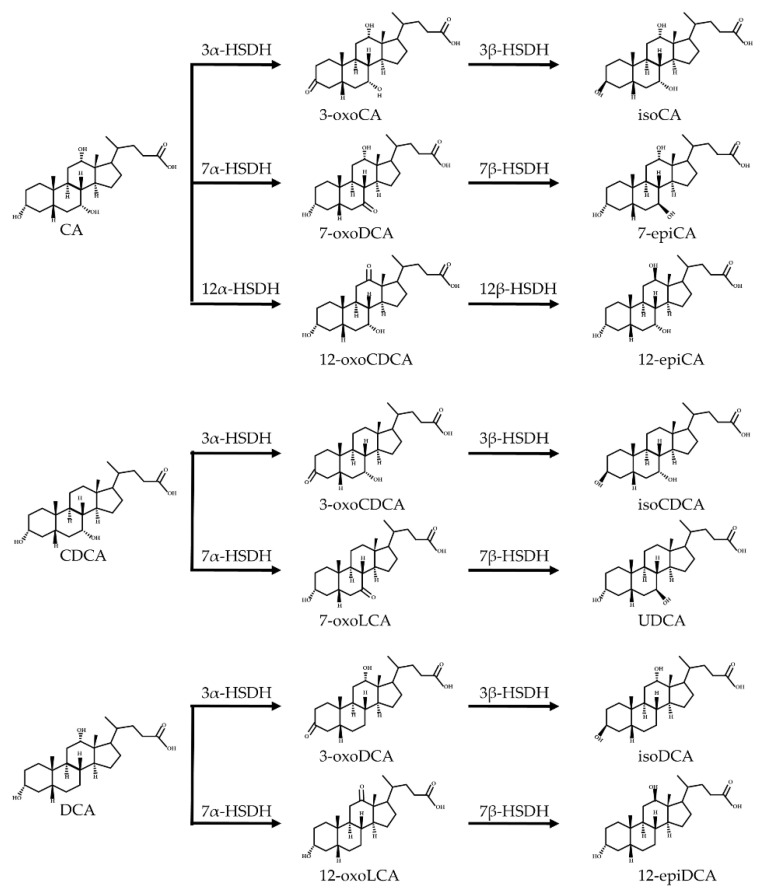
Oxidative and isomerization pathways of bile acids: Under the action of the α/β-hydroxy steroid dehydrogenase (HSDH) enzyme of the intestinal bacteria, primary bile acids produce oxygenated bile acid intermediates, which change the stereospecificity of bile acids, reduce their toxicity, and enrich the bile acid types.

Bacterial 3-, 7- and 12-hydroxysteroid dehydrogenases (HSDHs) catalyze the hydroxylation/isomerization of primary and secondary bile acids to their respective oxo-, epi-, and iso-bile acids. The isomerization of bile acid hydroxyl groups is a reversible stereo- chemical conversion from α to β configuration, resulting in stable oxygen-containing bile acid intermediates. The reduction and oxidation (REDOX) of bile acid hydroxyl groups largely depend on the REDOX potential of the mucosal environment. Mucosal surfaces with high REDOX potential are conducive to the production of oxygen-containing bile acids, while the intestinal lumen, with low REDOX potential, prefers to catalyze the reduction of oxygen-containing bile acids [43]. The oxidative isomerization pathway of bile acids is shown in Figure 4.

Specifically, 3α/β-HSDHs catalyze the reversible stereospecific oxidation/reduction between 3-oxygen bile acids and 3α- or 3β-hydroxyl bile acids, and the enzyme activities of 3α/β-HSDHs have been detected in some intestinal bacteria, including Firmicutes, *Digestive streptococcus*, and *Clostridium perfringens* [68]. Furthermore, 3α/β-HSDHs have different requirements for different pyridine nucleotide cofactors. While nicotinamide adenine dinucleotide (NAD(H)) is generally required for 3α-HSDHs, 3β-HSDHs prefer to count on nicotinamide adenine dinucleotide phosphate (NADP(H)) as their cofactor [43]. Di-hydroxy more than tri-hydroxy bile acids are prone to undergoing REDOX reactions. CA is converted to 3-oxo-CA and 3-iso-CA under the action of 3α-HSDH and 3β-HSDH, respectively. CDCA becomes 3-oxo-CDCA and 3-iso-CDCA, and DCA is converted to be 3-oxo-DCA by 3α-HSDH [69,70]. In contrast to α-hydroxyl isomers, iso-bile acids are more hydrophilic and less toxic to bacteria. For example, the isomerization of DCA is able to destroy the hydrophobic/hydrophilic surface properties of the bile acids, which may reduce the detergent properties and toxicity of DCA against bacteria, preventing DCA-induced DNA and membrane damage [62,68].

7α/β-HSDHs catalyze the reversible stereospecific oxidation/reduction between 7α- and 7β-hydroxyl bile acids. The 7α/β-HSDHs are widely expressed in *Clostridium*, *Eubacterium*, *Fusobacterium*, *Bacteroidetes*, and *Escherichia*, and prefer to use NADP(H) as a cofactor [71]. The affinity of 7α/β-HSDHs for di-hydroxy bile acids is higher than for tri-hydroxy bile acids. The conversions of CA into 7-oxo-DCA and CDCA into 7-oxo-LCA and UDCA by the 7α-HSDH and 7β-HSDH enzymes, respectively, produce the more hydrophilic intermediates and decrease toxicity against bacteria [66,69,72]. CDCA is more hydrophobic and toxic to bacteria than UDCA. Furthermore, for increasing the hydrophilicity of the bile acid pool and decreasing cytotoxicity, UDCA has been used as a veritable bile acid candidate in the treatment of biliary tract diseases [73,74].

The 12α/β-HSDHs are responsible for the reversible stereospecific oxidation/reduction reactions between 12α- and 12β-hydroxyl bile acids. *Clostridium* possesses 12α-HSDH and 12β-HSDH enzyme activities, requiring NAD(H) or NADP(H) as a cofactor [75]. The 12α-HSDHs reversibly convert bile acid at the C12 position from an α configuration to a 12-oxygen bile acid, and subsequently 12β-HSDHs convert 12-oxygen bile acid to a 12β configuration and consequently form epi-bile acids [41]. 12α-HSDH is responsible for the conversion of CA to 12-oxo-CDCA and 12-epi-CA, and of DCA to 12-oxoLCA, which is further transformed to be epi-DCA by 12β-HSDH.

#### 4.1.2. The Intestinal Flora Affects Compositions of the Bile Acid Pool

The intestinal flora enriches the diversity of bile acids and also modulates their synthesis and transportation. FXR is a nuclear receptor highly expressed in the gastrointestinal tract and liver. CDCA, CA, DCA, and LCA, along with their glycine and taurine-binding bile acids are agonists of FXR; UDCA and T-α/β-MCA are antagonists of FXR [76]. The presence of BSH in the intestinal flora reduces T-βMCA, which is an intestinal FXR antagonist. In hepatocytes, FXR induces SHP to inhibit *CYP7A1* and *CYP8B1* gene transcription, and bile acids enter the bile via BSEP and are reabsorbed into enterocytes. FXR induces IBABP and OSTα/β to expel bile acids from the portal circulation [77]. FXR also induces the expression of fibroblast growth factors (FGF15/FGF19), which travels to the liver via the portal vein, activating the FGFR4/β-Klotho heterodimer-JNK1/2 and ERK1/2 signaling pathways to subsequently inhibit the expression of CYP7A1 and bile acid synthesis [78]. On the other hand, ileal Kruppel-like factor 15 (KLF15) upregulates hepatic CYP7A1 and bile acid synthesis by inhibiting ileal production of FGF15 in mice [79]. In addition to suppressing bile acid synthesis, active FXR transactivates the expression of BSEP to stimulate bile acid secretion from the liver to the biliary duct but inhibits the expression of NTCP and reduces bile acid reabsorption from the blood to the liver [80]. Notably, intestinal flora regulation of bile acid transportation is mainly recognized through its regulatory effects on the bile acid reuptake transporter. While a small portion is reabsorbed via passive diffusion in the small intestine and colon, approximately 95% of bile acids are actively reabsorbed by ASBT, which is known to be regulated by the transcription factor GATA4 in intestinal epithelial cells. Intestinal bacteria can stimulate the expression of GATA4 and inhibit the expression of ABST, decreasing the reabsorption of bile acids by the ileum—the terminal region of the small intestine [81,82]. Studies have shown that bacteria are involved in the amino acid modification of bile acids in the intestine. In normal mice, bacteria can mediate the conjugation of bile acids with phenylalanine, tyrosine, and leucine, in addition to glycine and taurine, but only glycine- and taurine-conjugated bile acids can be detected in germ-free mice [83].

### 4.2. Effects of Bile Acids on the Intestinal Flora

#### 4.2.1. Bile Acids Inhibit the Growth and Proliferation of Bacteria

Bile acids can stimulate the growth of bacteria with bile-acid-metabolizing enzymes but inhibit the growth of bile-acid-sensitive bacteria, thereby maintaining the homeostasis of the intestinal flora and intestinal barrier function to prevent bacterial migration. Due to their stronger protective capacity, Gram-negative and other susceptible bacteria species are largely resistant to bile acid-induced membrane and DNA damage, and cell death [54]. The sensitive bacteria—such as *Spirochetes*, *Staphylococcus*, *Pneumococcus*, and *Enterococcus*—are subjected to bile-acid-induced membrane damage in a dose-dependent manner. Unconjugated bile acids often demonstrate stronger antibacterial ability than their conjugated counterparts [62,84]. Furthermore, the deficiency of bile acids in the intestine can cause excessive growth of bacteria and potent pathogens, increasing the risks for inflammation, bacterial translocation, membrane damage, hydrophobicity, and ion transmembrane flow [85]. Additionally, bile acids can directly bind to phospholipids in bacterial membranes and destroy the membrane structures, resulting in bacterial membrane damage and ultimately cell death [61,86]. Bile-acid-activated FXR negatively regulates bile acid synthesis and also induces iNOS and IL-18 to inhibit bacterial overgrowth and maintain the integrity of the intestinal epithelial layer [87].

#### 4.2.2. Bile Acids Affect the Composition of the Intestinal Flora

The effect of the intestinal flora on the composition of bile acids is not unidirectional, as bile acids can also affect the structure of the intestinal flora. CA can inhibit the growth of Gram-negative bacteria in the gastrointestinal tract and also potentiate the abundance of Firmicutes, such as *Clostridium XIVa*, along with bile acid 7α-dehydroxylase-containing bacteria [51,88]. DCA can inhibit Gram-positive bacteria, such as *Clostridium perfringens*, *Bacteroides fragilis*, *Lactobacillus*, and *Bifidobacteria*, mainly by destroying bacterial membrane integrity and causing bacterial death [89,90,91]. Long-term supplementation with exogenous DCA reduces BSH-containing bacteria and induces inflammation in murine intestines. DCA in the diet and gut dysbiosis can induce an imbalance in bile acid metabolisms, such as enhanced levels of TMCA, or suppression of the intestinal FXR-FGF15 pathway and therefore stimulate the *de novo* synthesis of bile acids in the liver [92]. In addition, UDCA can improve the imbalance in the intestinal flora by normalizing the ratio of Firmicutes to Bacteroidetes and stimulating the growth of BSH-rich bacteria, while accelerating the healing of the ulcerated epithelium. Further investigation revealed that UDCA has anti-inflammatory, anti-apoptotic, and anti-oxidative effects in the murine intestine [93,94]. IsoalloLCA has an inhibitory effect on the growth of Gram-positive bacteria, such as *Clostridioides difficile*, in the intestinal tract, and the germination of *Clostridioides difficile* spores in the intestine depends on the presence of endogenous bile acids but has no effect on Gram-negative bacteria [95]. A higher concentration of bile acids in the lumen is conducive to the growth of bacteria with 7α-dehydroxylase activity, whereas a lower concentration is mainly beneficial for the growth of Gram-negative bacteria. Bile acids also affect the integrity of intestinal epithelial cells and mucosal immune response, thereby indirectly regulating the composition of the microbial community [96,97].

### 4.3. Role of the Intestinal Flora and Bile Acids in Related Diseases

Under normal conditions, the intestinal flora builds up a dynamic balance with the host and the external environment. Interruption of this balance may cause various functional abnormalities in the host, such as an abnormal barrier function and immune function and inflammation, causing disease onset. Several diseases are closely related to the small metabolites (e.g., short-chain fatty acids and bile acids) dependent connective relationships between the intestinal flora and the host. As an important part of intestinal micro-ecology, the intestinal flora can directly participate in bile acid metabolism to affect biological functions and environmental homeostasis in the intestine. Any abnormality in bile acid metabolism may alter the community structure of the intestinal flora, which is closely related to the development of diseases, such as IBD, CRC, HCC, T2DM, and PCOS.

#### 4.3.1. Inflammatory Bowel Disease

IBD refers to the chronic and recurrent inflammatory diseases of the digestive tract, mainly including ulcerative colitis (UC) and Crohn’s disease [98]. Several factors including genetic and environmental factors and the state of intestinal micro-ecology play important roles in the pathogenesis of IBD. It is thought that IBD results from an abnormal and persistent immune response to the gut microbiome catalyzed by an individual’s genetic predisposition. Cytokines are produced and inflammatory cells are recruited, leading in turn to damage to the intestinal mucosal barrier, often taking the form of ulcers [99]. Micro-ecological imbalance is also a typical inducer for the pathogenesis of IBD. Intestinal ecological imbalance causes an intestinal immune response, along with dysregulations in lipopolysaccharide (LPS), teichoic acid, and bile acids, which are crucial events in the process of IBD’s development [100].

Ecological imbalance of the intestinal flora in IBD patients results in a poor bacterial diversity, including an enhanced proportion of Bacteroidetes but a reduction in Firmicutes. At the genus level, the proportion of *Bacteroides* was found to increase while the proportion of *Clostridium XIVa* was found to decrease [101,102]. While harmful bacteria such as Proteobacteria and Actinobacteria increased, bacteria such as *Ruminococcus*, *Lachnospira*, and *Eubacterium*, which were enriched in BSH and HSDH, decreased [103]. Moreover, a bacterial ecological imbalance in IBD patients leads to reducing the abundance of intestinal secondary bile acids (e.g., DCA, LCA, and TLCA) and the enhancement of primary bile acids (e.g., CA, CDCA, GCA, and TCA) [104]. Studies have shown that DCA-fed mice induce the release of inflammatory factors and the imbalance of the intestinal flora, leading to a disorder in bile acid metabolism in the liver and the small intestine [92]. Increased DCA and TMCA down-regulate the FXR-FGF15 signaling pathway and inhibit the expression of OSTα but promote the expression of CYP7A1 and bile acid synthesis. These effects potentially destroy the enterohepatic circulation of bile acids and also promote intestinal inflammation.

Intestinal dysbiosis and disorders in bile acid metabolism promote the development of IBD; thus, the reordering intervention of the intestinal flora and bile acids may be an effective strategy for the treatment of IBD. Notably, fecal microbiota transplantation (FMT) is a treatment for restoring the composition of the intestinal microbiome by transplanting fecal microorganisms obtained from healthy donors into patients [99]. FMT treatment for IBD has yielded appreciably positive effects, especially in UC [100]. While FMT treatment is mostly safe, a small number of patients have experienced infection and death after the treatment. Therefore, further studies are needed to prove the reliability of FMT treatment for IBD [105]. Interestingly, supplementary treatment with probiotics ensures the improvement of the intestinal flora, enhancement of intestinal barrier function, a reduction in intestinal inflammation, and a general relief of symptoms in IBD patients [106]. Furthermore, studies have shown that FXR agonists can reduce DCA-induced intestinal injury, restore the intestinal FXR activity of transactivating FGF15, normalize bile acid metabolism, and restore the intestinal microbiota [107]. Moreover, treating IBD mice with UDCA, TUDCA, and GUDCA can reduce intestinal dysbiosis and improve inflammation, restore the ratio of Firmicutes to Bacteroidetes in the intestine, increase the abundance of BSH-rich bacteria, and reduce intestinal proinflammatory cytokine (e.g., IL-1β and IL-6) levels, accelerating the healing of the ulcerated epithelium and protecting colon cells from apoptosis and oxidative damage, making UDCA a viable therapeutic option [108].

#### 4.3.2. Colorectal Cancer

CRC is a heterogeneous disease of the intestinal epithelium and one of the most common malignant tumors, ranking third globally in terms of mortality [109]. The occurrence and the development of CRC involves complex interactions among genetic, epigenetic, and environmental factors, including CRC triggered by environmental factors or stunted adenomatous polyps [110]. Intestinal dysbiosis is one of the most important environmental factors facilitating the development of CRC. Essentially, the metabolites produced by the intestinal flora can cause colon inflammation and the development of CRC.

In CRC patients, the intestinal flora is misbalanced, in that operational taxonomic units (OTUs) belonging to particular genera of gut bacteria are unevenly distributed in the gut. Genera of the types *Clostridium nuclear*, *Escherichia coli*, *Bacteroides fragilis*, *Enterococcus*, *Streptococcus*, and *digestive Streptococcus* are highly abundant; and, the numbers of *Bacteroides*, *Rothia*, *Clostridium*, *Ruminococcus*, *Eubacterium*, *Lactobacillus*, and *Bifidobacteria* are reduced [111]. Studies have shown that the intestinal-microbial-produced secondary bile acid, DCA, contributes to the development of colorectal cancer by disrupting intestinal barrier function, promoting the recruitment of tumor-associated macrophages, and stimulating tumor cell proliferation through the activation of the Wnt/β-catenin signaling pathway in mutation-susceptible murine models [112]. DCA, at low concentrations, promotes the growth and metastasis of colon cancer by activating the β-catenin-cyclin D1 and -uPAR signaling pathways. Additionally, DCA has been reported to mediate the transactivation of EGFR, the phosphorylation of P44/42 MAPK, and the activation of downstream transcription factors that stimulate cell proliferation [113,114]. Collectively, the processes above result in the promotion and the development of CRC.

Interestingly, *Clostridium butyricum*—a butyricum producing probiotic—has been shown to inhibit the development of HFD-induced intestinal tumors in Apc^min/+^ mice, mainly by down-regulating the Wnt/β-catenin signaling pathway to inhibit the proliferation of tumor cells but stimulating their apoptosis. Since *Clostridium butyricum* can partially rescue the ecological imbalance of the intestinal microbiota, the levels of microbial metabolites, such as short-chain fatty acids and bile acids, tend to be normal, preventing the pathogenesis of CRC [115]. Notably, probiotics produce antimicrobial peptides or reduce the intestinal lumen’s pH to inhibit the colonization of pathogens, or promote epithelial recovery and enhance intestinal barrier function by increasing mucin production and tight-junction protein expression. FOLFOX (5-fluorouracil, leucovorin, and oxaliplatin) is one of the most common therapeutic regimens for CRC, but it can cause adverse effects, such as intestinal mucositis. The efficacy and the safety of FMT in cancer patients treated with antineoplastic drugs are still insufficient. FMT reduces the severity of FOLFOX-induced diarrhea and intestinal mucosa and also restores the composition of the gut microbiota [116]. Importantly, imbalances or dysbiosis in the intestinal flora and bile acids play important roles in the development of CRC, and they can be used as biomarkers to predict therapeutic efficacy and for improving cancer treatment through the adjustment of the intestinal flora [117].

#### 4.3.3. Hepatocellular Carcinoma

HCC is the fourth leading cause of death from cancer, and it has a high mortality rate. The development of liver cancer includes the stages of chronic inflammatory liver disease, hepatitis, liver fibrosis, and liver tumorigenesis [118]. In non-viral HCC, intestinal barrier dysfunction associated with chronic liver diseases due to a high-fat diet, alcohol, or increased levels of secondary bile acids, allows for the entry of intestinal microbiota products into the liver, activating hepatic stellate cells (HSCs) and Toll-like receptor 4 (TLR4), and the concomitant induction of fibrosis and the epithelium-regulated protein growth factor secretion, ultimately promoting the development of liver cancer [119,120].

Furthermore, observations of intestinal ecological disorders in HCC patients are accompanied by increased levels of *Clostridium*, *Veillonella*, *Enterobacteriaceae*, and *Fusobacterium* and reductions in the abundance of butyrate-producing *Ruminococcus* and *Lachnospira* [121]. In mice with NASH-HCC induced by streptozotocin and a high-fat diet (STZ–HFD), the hydrophobic bile acids (e.g., DCA, TCA, TCDCA, and TLCA) were significantly increased in the liver, the intestinal microflora were changed, and the genes involved in bile acid synthesis and transportation (e.g., CYP7A1, BSEP) were down-regulated. The increased expression of the pro-inflammatory genes IL-6 and TNF-α was found to contribute to the development of HCC [122]. Leaky gut leads to high circulating levels of MAMPs, such as LPS in multiple stages of hepatocarcinogenesis. LPS and its receptor, TLR, induce hepatocarcinogenesis, and TLR4 also promotes the development of liver fibrosis in hepatic stellate cells. Metabolites of intestinal flora derivatives, such as DCA, increase the expression of TLR2 in hepatic stellate cells, resulting in increased levels of the TLR2 agonist lipoteichoic acid [123].

Importantly, understanding the relationship between the development of HCC and bile acid–intestinal flora bidirectional interaction, makes it possible to improve the microbiome and restore intestinal homeostasis in future therapeutic interventions. Studies have shown that probiotics can reduce the occurrence of HCC induced by DEN; protect the integrity of the intestinal mucosa; reduce intestinal permeability, and the levels of plasma LPS and the inflammatory cytokine IL-6; and restore intestinal homeostasis to resist liver inflammation [124]. Moreover, probiotics are able to inhibit tumor angiogenesis, resist ecological dysregulation, promote the establishment of non-inflammatory bacteria, change the composition of a dysregulated microbiome, and reduce liver inflammation and the development of HCC [125]. Studies have shown that the removal of Gram-positive bacteria by antibiotic treatment with vancomycin—including bacteria that mediate primary-to-secondary bile acid conversion—is sufficient to induce hepatic NKT cell accumulation and reduce liver tumor growth [123,126]. Feeding secondary bile acids or colonization with bile-acid-metabolizing bacteria reverses NKT cell accumulation and liver tumor growth inhibition in mice with altered gut commensal bacteria. Finally, FMT has become a useful method for the alteration of the intestinal microbiome in some cases, and its therapeutic effect in liver cancer needs further investigation [127].

#### 4.3.4. Type 2 Diabetes

T2DM is a chronic metabolic disease characterized by hyperglycemia, mainly due to insulin resistance and insufficient insulin secretion [128]. With an increasing incidence worldwide, T2DM is caused by multiple factors, including genetic predisposition, age, obesity, and other unhealthy lifestyles. Intestinal microbial metabolites and bacterial components affect the occurrence and the development of T2DM by regulating inflammatory response, immunity, and metabolism.

In T2DM patients, the abundances of *Bifidobacteria*, *Clostridium*, and Firmicutes are reduced, but the abundances of Bacteroidetes and β-proteobacteria are increased. Moreover, the ratio of Bacteroidetes to Firmicutes and the ratio of Firmicutes to *Clostridium*, are found to be proportional to blood glucose levels [129]. Furthermore, the intestinal permeability and the circulating levels of endotoxins from *Bifidobacterium*, *Ruminococcus*, and *Rothia* were all found to be increased. Interestingly, dysregulation of the intestinal flora alters the levels of bile acid metabolites: most conjugated bile acids—such as TCDCA, TDCA, GDCA, and HDCA—increase [130]. Decreased activation of FXR reduces both insulin sensitivity and glycogen synthesis; increases hepatic gluconeogenesis and blood glucose; and also decreases FGF19, FGF21, and energy expenditure [131]. LPS has also been found to activate the TLR4 receptor, trigger the release of inflammatory factors, promote apoptosis of islet B cells, and induce insulin resistance, leading to T2DM [132].

In terms of treatment strategies, metformin is currently being used as a first-line hypoglycemic agent for T2DM. This drug increases intestinal flora content and composition, the excretion of fecal bile acids, and clP-1 secretion but reduces bile acid reabsorption and plasma glucose levels [133,134]. Intestinal microbiota composition is closely associated with the occurrence of T2DM; thus, its functional improvement may be a therapeutic strategy and an intervention target for diabetes. Impressively, probiotics can improve the intestinal microbiota and peripheral insulin sensitivity but reduce LPS levels and endoplasmic reticulum stress. Mice treated with FMT show improved insulin sensitivity and an increased abundance of butyrate-producing bacteria. However, further studies are needed to further assess the long-term efficacy and potent side effects of FMT treatment [135].

#### 4.3.5. Polycystic Ovary Syndrome

PCOS is a disease characterized by androgen excess, ovulation dysfunction, and morphological characteristics of polycystic ovary, and it is the most common endocrine disease in women of childbearing age. Its prevalence rate is up to 10% [136]. Studies have shown that PCOS is influenced by genetic and environmental factors, and it has a long-term association with metabolic disorders, obesity, and T2DM [137].

The intestinal flora and bile acids of patients with PCOS are significantly different from those of healthy people. Specifically, *Lactobacilli*, *Streptococcus*, and *Escherichia coli* are higher in PCOS patients; and *Ruminococcus*, *Lachnospiraceae*, and *Prevotella* are lower [138,139,140]. Concisely, bile acids affect the regulation of ovarian cell function, causing ovulation dysfunction in the patients [141]. In comparison with healthy individuals, the primary conjugated bile acids GCA, TCA, and GCDCA were detected at higher levels in PCOS patients [142]. Moreover, intestinal ecological imbalance and decreases in TDCA and GDCA levels have been found to be associated with a reduction in the activity of transcription factor GATA-binding protein 3, thereby reducing the secretion of IL-22 by intestinal type-3 natural lymphocytes. The consequent elevation of brown fat has been observed to inhibit the inflammatory response of ovarian granulosa cells, modulating ovarian function and insulin sensitivity in polycystic ovary syndrome [143]. The endotoxic effect of LPS produced by the intestinal flora has also been studied. Briefly, LPS, after entering the blood, activates the TLR4 receptor, resulting in increased expression of TNF-α and IL-6, which in turn induce insulin resistance and an inflammatory response, thereby promoting the occurrence and development of PCOS [144].

In summary, the intestinal flora may play a role in the development of PCOS and many of its clinical symptoms. Thus, intestinal bacteria serve as potential therapeutic targets. Importantly, probiotics can maintain the intestinal flora, improve intestinal permeability, prevent bacterial translocation from the intestinal tract, and reduce inflammation [145]. Notwithstanding the lack of clinical reports about FMT for the treatment of gynecological diseases, studies in rat models have found that FMT can significantly improve symptoms in PCOS rats, essentially including increasing androgen levels and significantly reducing estradiol and estrone levels to appreciably normalize ovarian function [146].

## 5. Concluding Remarks

The interaction between intestinal flora and bile acids is bidirectional, mutually beneficial, and critical for the maintenance of normal physiology. While intestinal flora affects the synthesis, metabolism, and composition of bile acids, the latter, on the other hand, regulate the diversity and the structure of intestinal flora. Under physiological conditions, bile acids and intestinal flora are in a dynamic balance. Homeostatic imbalances between intestinal flora and bile acids are involved in the development of diseases, including IBD, HCC, PCOS, T2DM, and CRC. The accumulation of secondary bile acid, DCA, plays a central role in the development of these diseases as it induces damage in cells and also causes an increase in harmful bacteria but decreases the abundance of beneficial bacteria, respectively. In terms of treatment strategy, the type or quantity of bile acids in patients can be changed to reduce inflammation, and the composition of the intestinal flora can be transplanted or improved by supplementing beneficial bacteria to alleviate the progression of diseases. UDCA increases the hydrophilicity of the bile acid pool and protects cells against apoptosis. Furthermore, FMT can also be used for treating a variety of diseases caused by intestinal flora–bile acid homeostatic imbalances. Taken together, bile acids and the intestinal flora play important roles in human health. The maintenance of the homeostatic balance between the intestinal flora and bile acids is critical for the performance of their normal physiological functions in various metabolic pathways and for the prevention of the onset of related pathologies. Gut microbiota and bile acids as novel targets for therapies, as well as for disease control and prevention research, can include beneficial commensal microbiota and their key metabolites, which control the type and the quantity of bile acids, along with their involvement in bile acid synthesis, transport, and metabolic signaling pathways, and they may become a new research frontier.

## Figures and Tables

**Table 1:** Classification of bacterial species in the intestinal flora: According to classification by natural properties, intestinal bacteria can be divided into six categories for the most part: Firmicutes, Bacteroidetes, Proteobacteria, Actinobacteria, Fusobacteria, and Verrucomicrobia. Each category includes bacterial species.

Phylum	Class	Order	Family	Genus	Species
Firmicutes	Clostridia	Clostridiales	Clostridiaceae	*Faecalibacterium*	*Faecalibacterium prausnitzii*
				*Clostridium*	*Clostridium* spp.
			Lachnospiraceae	*Coprocococcus*	*Coprococcus eutactus*
			Peptostreptococcaceae	*Peptostreptococcus*	*Peptostreptococcus anaerobius*
			Veillonellaceae	*Veillonella*	*Veillonella parvula*
	Bacilli	Lactobacillales	Lactobacillaceae	*Lactobacillus*	*Lactobacillus acidophilus*
			Enterococcaceae	*Enterococcus*	*Enterococcus faecalis*
		Bacillales	Listeriaceae	*Listeria*	*Listeria iuanuii*
Bacteroidetes	Flavobacteria	Flavobacteriales	Flavobacteriaceae	*Flavobacterium*	
	Bacteroidetes	Bacteroidales	Bacteroidaceae	*Bacteroides*	*Bacteroides fragilis*
					*Bacteroides caccae*
					*Bacteroides pyogenes*
			Porphyromonadaceae	*Porphyromonas*	
				*Parabacteroides*	*Parabacteroides distasonis*
			Rikenellaceae	*Alistipes*	*Alistipes finegoldii*
			Prevotellaceae	*Prevotella*	*Prevotella* spp.
Proteobacteria	Gamma proteobacteria	Enterobacteriales	Enterobacteriaceae	*Escherichia*	*Escherichia coli*
				*Enterobacter*	*Enterobacter areogenes*
	Delta proteobacteria	Desulfovibrionales Desulfobacterales	Desulfovibrionaceae Desulfobacteraceae	*Desulfovibrio*	*Desulfovibrio intestinalis*
				*Desulfobacter*	
	Epsilon proteobacteria	Campylobacterales	Helicobacteraceae	*Helicobacter*	*Helicobacter pylori*
Actinobacteria	Actinobacteria	Actinomycetales	Actinomycetaceae	*Actinobaculum*	
			Corynebacteriaceae	*Corynebacterium*	*Corynebacterium glutamicum*
		Bifidobacteriales	Bifidobacteriaceae	*Bifidobacterium*	*Bifidobacterium adolescentis*
					*Bifidobacterium longum*
Fusobacteria	Fusobacteria	Fusobacteriales	Fusobacteriaceae	*Fusobacterium*	*Fusobacterium nucleatum*
Verrucomicrobia	Verrucomicrobiae	Verrucomicrobiales	Verrucomicrobiaceae	*Akkermansia*	*Akkermansia muciniphila*

**Table 2:** The structures of bile acids: The structural formula of bile acids and bile acid intermediates along with the hydroxyl positions are presented below, and the hydrophobicity of bile acid can be assessed according to their stereoscopic configurations.

Structural Formula	Abbreviation	C3	C6	C7	C12
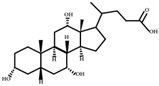	CA	3α-OH	H	7α-OH	12α-OH
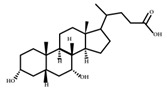	CDCA	3α-OH	H	7α-OH	H
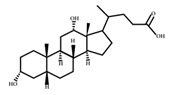	DCA	3α-OH	H	H	12α-OH
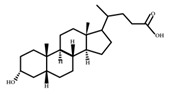	LCA	3α-OH	H	H	H
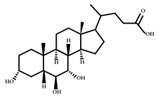	α-MCA	3α-OH	6β-OH	7α-OH	H
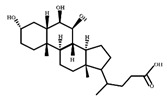	β-MCA	3α-OH	6β-OH	7β-OH	H
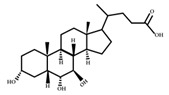	ω-MCA	3α-OH	6α-OH	7β-OH	H
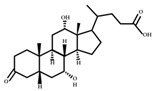	3-oxoCA	oxo	H	7α-OH	12α-OH
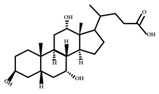	IsoCA	3β-OH	H	7α-OH	12α-OH
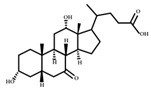	7-oxoDCA	3α-OH	H	oxo	12α-OH
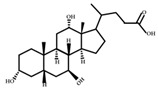	7-epiCA	3α-OH	H	7β-OH	12α-OH
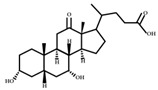	12-oxoCDCA	3α-OH	H	7α-OH	oxo
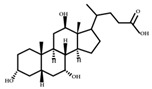	12-epiCA	3α-OH	H	7α-OH	12β-OH
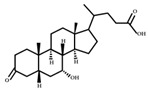	3-oxoCDCA	oxo	H	7α-OH	H
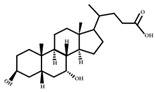	IsoCDCA	3β-OH	H	7α-OH	H
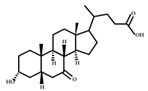	7-oxoLCA	3α-OH	H	oxo	H
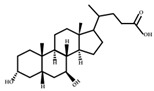	UDCA	3α-OH	H	7β-OH	H
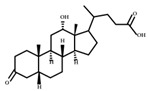	3-oxoDCA	oxo	H	H	12α-OH
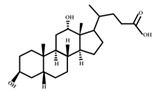	IsoDCA	3β-OH	H	H	12α-OH
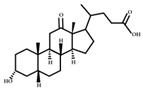	12-oxoLCA	3α-OH	H	H	oxo
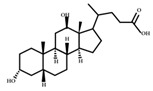	EpiDCA	3α-OH	H	H	12β-OH
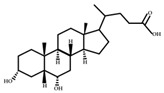	HDCA	3α-OH	6α-OH	H	H
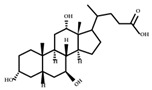	UCA	3α-OH	H	7β-OH	12α-OH
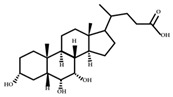	HCA	3α-OH	6α-OH	7α-OH	H

## Data Availability

Not applicable.

## References

[1] Anwar H., Iftikhar A., Muzaffar H., Almatroudi A., Allemailem K.S., Navaid S., Saleem S., Khurshid M. (2021). Biodiversity of Gut Microbiota: Impact of Various Host and Environmental Factors. BioMed Res. Int..

[2] Zhang Y.J., Li S., Gan R.Y., Zhou T., Xu D.P., Li H.B. (2015). Impacts of gut bacteria on human health and diseases. Int. J. Mol. Sci..

[3] Albhaisi S.A.M., Bajaj J.S., Sanyal A.J. (2020). Role of gut microbiota in liver disease. Am. J. Physiol. Gastrointest. Liver Physiol..

[4] Shi N., Li N., Duan X., Niu H. (2017). Interaction between the gut microbiome and mucosal immune system. Mil. Med. Res..

[5] Lavelle A., Sokol H. (2020). Gut microbiota-derived metabolites as key actors in inflammatory bowel disease. Nat. Rev. Gastroenterol. Hepatol..

[6] Singh J., Metrani R., Shivanagoudra S.R., Jayaprakasha G.K., Patil B.S. (2019). Review on Bile Acids: Effects of the Gut Microbiome, Interactions with Dietary Fiber, and Alterations in the Bioaccessibility of Bioactive Compounds. J. Agric. Food Chem..

[7] Di Gregorio M.C., Cautela J., Galantini L. (2021). Physiology and Physical Chemistry of Bile Acids. Int. J. Mol. Sci..

[8] Rodriguez-Morato J., Matthan N.R. (2020). Nutrition and Gastrointestinal Microbiota, Microbial-Derived Secondary Bile Acids, and Cardiovascular Disease. Curr. Atheroscler. Rep..

[9] Ticho A.L., Malhotra P., Dudeja P.K., Gill R.K., Alrefai W.A. (2019). Intestinal Absorption of Bile Acids in Health and Disease. Compr. Physiol..

[10] Jia B., Park D., Hahn Y., Jeon C.O. (2020). Metagenomic analysis of the human microbiome reveals the association between the abundance of gut bile salt hydrolases and host health. Gut Microbes.

[11] Milani C., Ferrario C., Turroni F., Duranti S., Mangifesta M., van Sinderen D., Ventura M. (2016). The human gut microbiota and its interactive connections to diet. J. Hum. Nutr. Diet..

[12] Tanaka M., Sanefuji M., Morokuma S., Yoden M., Momoda R., Sonomoto K., Ogawa M., Kato K., Nakayama J. (2020). The association between gut microbiota development and maturation of intestinal bile acid metabolism in the first 3 y of healthy Japanese infants. Gut Microbes.

[13] Adlerberth I., Wold A.E. (2009). Establishment of the gut microbiota in Western infants. Acta Paediatr..

[14] Durack J., Lynch S.V. (2019). The gut microbiome: Relationships with disease and opportunities for therapy. J. Exp. Med..

[15] Hollister E.B., Riehle K., Luna R.A., Weidler E.M., Rubio-Gonzales M., Mistretta T.A., Raza S., Doddapaneni H.V., Metcalf G.A., Muzny D.M. (2015). Structure and function of the healthy pre-adolescent pediatric gut microbiome. Microbiome.

[16] Adak A., Khan M.R. (2019). An insight into gut microbiota and its functionalities. Cell Mol. Life Sci..

[17] Gomaa E.Z. (2020). Human gut microbiota/microbiome in health and diseases: A review. Antonie Van Leeuwenhoek.

[18] Gonzalez Olmo B.M., Butler M.J., Barrientos R.M. (2021). Evolution of the Human Diet and Its Impact on Gut Microbiota, Immune Responses, and Brain Health. Nutrients.

[19] Dewhirst F.E., Chen T., Izard J., Paster B.J., Tanner A.C., Yu W.H., Lakshmanan A., Wade W.G. (2010). The human oral microbiome. J. Bacteriol..

[20] Wade W.G. (2013). The oral microbiome in health and disease. Pharmacol. Res..

[21] Martinez-Guryn K., Leone V., Chang E.B. (2019). Regional Diversity of the Gastrointestinal Microbiome. Cell Host Microbe.

[22] Pei Z., Bini E.J., Yang L., Zhou M., Francois F., Blaser M.J. (2004). Bacterial biota in the human distal esophagus. Proc. Natl. Acad. Sci. USA.

[23] Jandhyala S.M., Talukdar R., Subramanyam C., Vuyyuru H., Sasikala M., Nageshwar Reddy D. (2015). Role of the normal gut microbiota. World J. Gastroenterol..

[24] Nardone G., Compare D. (2015). The human gastric microbiota: Is it time to rethink the pathogenesis of stomach diseases?. United Eur. Gastroenterol. J..

[25] O’Hara A.M., Shanahan F. (2006). The gut flora as a forgotten organ. EMBO Rep..

[26] Weaver K.E. (2019). Enterococcal Genetics. Microbiol. Spectr..

[27] Ma Q., Li Y., Li P., Wang M., Wang J., Tang Z., Wang T., Luo L., Wang C., Wang T. (2019). Research progress in the relationship between type 2 diabetes mellitus and intestinal flora. Biomed. Pharmacother..

[28] Krawczyk B., Wityk P., Galecka M., Michalik M. (2021). The Many Faces of Enterococcus spp.-Commensal, Probiotic and Opportunistic Pathogen. Microorganisms.

[29] Murphy E.C., Frick I.M. (2013). Gram-positive anaerobic cocci—Commensals and opportunistic pathogens. FEMS Microbiol. Rev..

[30] Martin R.M., Bachman M.A. (2018). Colonization, Infection, and the Accessory Genome of Klebsiella pneumoniae. Front. Cell Infect. Microbiol..

[31] Chiang J.Y. (2013). Bile acid metabolism and signaling. Compr. Physiol..

[32] Thomas C., Pellicciari R., Pruzanski M., Auwerx J., Schoonjans K. (2008). Targeting bile-acid signalling for metabolic diseases. Nat. Rev. Drug Discov..

[33] De Aguiar Vallim T.Q., Tarling E.J., Edwards P.A. (2013). Pleiotropic roles of bile acids in metabolism. Cell Metab..

[34] Styles N.A., Shonsey E.M., Falany J.L., Guidry A.L., Barnes S., Falany C.N. (2016). Carboxy-terminal mutations of bile acid CoA:N-acyltransferase alter activity and substrate specificity. J. Lipid Res..

[35] Jia W., Xie G., Jia W. (2018). Bile acid-microbiota crosstalk in gastrointestinal inflammation and carcinogenesis. Nat. Rev. Gastroenterol. Hepatol..

[36] Chen M.L., Takeda K., Sundrud M.S. (2019). Emerging roles of bile acids in mucosal immunity and inflammation. Mucosal Immunol..

[37] Joyce S.A., Gahan C.G. (2016). Bile Acid Modifications at the Microbe-Host Interface: Potential for Nutraceutical and Pharmaceutical Interventions in Host Health. Annu. Rev. Food Sci. Technol..

[38] Shiffka S.J., Kane M.A., Swaan P.W. (2017). Planar bile acids in health and disease. Biochim. Biophys. Acta Biomembr..

[39] Thompson R.S., Gaffney M., Hopkins S., Kelley T., Gonzalez A., Bowers S.J., Vitaterna M.H., Turek F.W., Foxx C.L., Lowry C.A. (2021). Ruminiclostridium 5, Parabacteroides distasonis, and bile acid profile are modulated by prebiotic diet and associate with facilitated sleep/clock realignment after chronic disruption of rhythms. Brain Behav. Immun..

[40] Russell D.W. (2003). The enzymes, regulation, and genetics of bile acid synthesis. Annu. Rev. Biochem..

[41] Doden H.L., Wolf P.G., Gaskins H.R., Anantharaman K., Alves J.M.P., Ridlon J.M. (2021). Completion of the gut microbial epi-bile acid pathway. Gut Microbes.

[42] Busnelli M., Manzini S., Chiesa G. (2019). The Gut Microbiota Affects Host Pathophysiology as an Endocrine Organ: A Focus on Cardiovascular Disease. Nutrients.

[43] Ridlon J.M., Kang D.J., Hylemon P.B. (2006). Bile salt biotransformations by human intestinal bacteria. J. Lipid Res..

[44] Ridlon J.M., Devendran S., Alves J.M., Doden H., Wolf P.G., Pereira G.V., Ly L., Volland A., Takei H., Nittono H. (2020). The ‘in vivo lifestyle’ of bile acid 7alpha-dehydroxylating bacteria: Comparative genomics, metatranscriptomic, and bile acid metabolomics analysis of a defined microbial community in gnotobiotic mice. Gut Microbes.

[45] Li J., Dawson P.A. (2019). Animal models to study bile acid metabolism. Biochim. Biophys. Acta Mol. Basis Dis..

[46] Wahlstrom A., Sayin S.I., Marschall H.U., Backhed F. (2016). Intestinal Crosstalk between Bile Acids and Microbiota and Its Impact on Host Metabolism. Cell Metab..

[47] Yang Y., Zhang J. (2020). Bile acid metabolism and circadian rhythms. Am. J. Physiol. Gastrointest. Liver Physiol..

[48] Chiang J.Y. (2003). Bile acid regulation of hepatic physiology: III. Bile acids and nuclear receptors. Am. J. Physiol. Gastrointest. Liver Physiol..

[49] Xie G., Jiang R., Wang X., Liu P., Zhao A., Wu Y., Huang F., Liu Z., Rajani C., Zheng X. (2021). Conjugated secondary 12alpha-hydroxylated bile acids promote liver fibrogenesis. eBioMedicine.

[50] Jia W., Wei M., Rajani C., Zheng X. (2021). Targeting the alternative bile acid synthetic pathway for metabolic diseases. Protein Cell.

[51] Winston J.A., Theriot C.M. (2020). Diversification of host bile acids by members of the gut microbiota. Gut Microbes.

[52] Sayin S.I., Wahlstrom A., Felin J., Jantti S., Marschall H.U., Bamberg K., Angelin B., Hyotylainen T., Oresic M., Backhed F. (2013). Gut microbiota regulates bile acid metabolism by reducing the levels of tauro-beta-muricholic acid, a naturally occurring FXR antagonist. Cell Metab..

[53] Long S.L., Gahan C.G.M., Joyce S.A. (2017). Interactions between gut bacteria and bile in health and disease. Mol. Aspects Med..

[54] Staley C., Weingarden A.R., Khoruts A., Sadowsky M.J. (2017). Interaction of gut microbiota with bile acid metabolism and its influence on disease states. Appl. Microbiol. Biotechnol..

[55] Xiao Y., Zhao J., Zhang H., Zhai Q., Chen W. (2021). Mining genome traits that determine the different gut colonization potential of Lactobacillus and Bifidobacterium species. Microb. Genom..

[56] Foley M.H., O’Flaherty S., Allen G., Rivera A.J., Stewart A.K., Barrangou R., Theriot C.M. (2021). Lactobacillus bile salt hydrolase substrate specificity governs bacterial fitness and host colonization. Proc. Natl. Acad. Sci. USA.

[57] Enright E.F., Griffin B.T., Gahan C.G.M., Joyce S.A. (2018). Microbiome-mediated bile acid modification: Role in intestinal drug absorption and metabolism. Pharmacol. Res..

[58] Begley M., Gahan C.G., Hill C. (2005). The interaction between bacteria and bile. FEMS Microbiol. Rev..

[59] Daly J.W., Keely S.J., Gahan C.G.M. (2021). Functional and Phylogenetic Diversity of BSH and PVA Enzymes. Microorganisms.

[60] Chand D., Avinash V.S., Yadav Y., Pundle A.V., Suresh C.G., Ramasamy S. (2017). Molecular features of bile salt hydrolases and relevance in human health. Biochim. Biophys. Acta Gen. Subj..

[61] Bustos A.Y., Font de Valdez G., Fadda S., Taranto M.P. (2018). New insights into bacterial bile resistance mechanisms: The role of bile salt hydrolase and its impact on human health. Food Res. Int..

[62] Guzior D.V., Quinn R.A. (2021). Review: Microbial transformations of human bile acids. Microbiome.

[63] Funabashi M., Grove T.L., Wang M., Varma Y., McFadden M.E., Brown L.C., Guo C., Higginbottom S., Almo S.C., Fischbach M.A. (2020). A metabolic pathway for bile acid dehydroxylation by the gut microbiome. Nature.

[64] Mallonee D.H., Hylemon P.B. (1996). Sequencing and expression of a gene encoding a bile acid transporter from *Eubacterium* sp. strain VPI 12708. J. Bacteriol..

[65] Heinken A., Ravcheev D.A., Baldini F., Heirendt L., Fleming R.M.T., Thiele I. (2019). Systematic assessment of secondary bile acid metabolism in gut microbes reveals distinct metabolic capabilities in inflammatory bowel disease. Microbiome.

[66] Ridlon J.M., Harris S.C., Bhowmik S., Kang D.J., Hylemon P.B. (2016). Consequences of bile salt biotransformations by intestinal bacteria. Gut Microbes.

[67] Mallonee D.H., Adams J.L., Hylemon P.B. (1992). The bile acid-inducible baiB gene from Eubacterium sp. strain VPI 12708 encodes a bile acid-coenzyme A ligase. J. Bacteriol..

[68] Devlin A.S., Fischbach M.A. (2015). A biosynthetic pathway for a prominent class of microbiota-derived bile acids. Nat. Chem. Biol..

[69] Fiorucci S., Distrutti E. (2015). Bile Acid-Activated Receptors, Intestinal Microbiota, and the Treatment of Metabolic Disorders. Trends Mol. Med..

[70] Liu H.X., Keane R., Sheng L., Wan Y.J. (2015). Implications of microbiota and bile acid in liver injury and regeneration. J. Hepatol..

[71] Ferrandi E.E., Bertolesi G.M., Polentini F., Negri A., Riva S., Monti D. (2012). In search of sustainable chemical processes: Cloning, recombinant expression, and functional characterization of the 7α- and 7β-hydroxysteroid dehydrogenases from Clostridium absonum. Appl. Microbiol. Biotechnol..

[72] Lepercq P., Gerard P., Beguet F., Raibaud P., Grill J.P., Relano P., Cayuela C., Juste C. (2004). Epimerization of chenodeoxycholic acid to ursodeoxycholic acid by Clostridium baratii isolated from human feces. FEMS Microbiol. Lett..

[73] Goossens J.F., Bailly C. (2019). Ursodeoxycholic acid and cancer: From chemoprevention to chemotherapy. Pharmacol. Ther..

[74] Zhang X., Fan D., Hua X., Zhang T. (2019). Large-scale production of ursodeoxycholic acid from chenodeoxycholic acid by engineering 7alpha- and 7beta-hydroxysteroid dehydrogenase. Bioprocess. Biosyst. Eng..

[75] Doden H., Sallam L.A., Devendran S., Ly L., Doden G., Daniel S.L., Alves J.M.P., Ridlon J.M. (2018). Metabolism of Oxo-Bile Acids and Characterization of Recombinant 12alpha-Hydroxysteroid Dehydrogenases from Bile Acid 7alpha-Dehydroxylating Human Gut Bacteria. Appl. Environ. Microbiol..

[76] Sun L., Cai J., Gonzalez F.J. (2021). The role of farnesoid X receptor in metabolic diseases, and gastrointestinal and liver cancer. Nat. Rev. Gastroenterol. Hepatol..

[77] Chiang J.Y.L., Ferrell J.M. (2020). Bile acid receptors FXR and TGR5 signaling in fatty liver diseases and therapy. Am. J. Physiol. Gastrointest. Liver Physiol..

[78] Li Y., Tang R., Leung P.S.C., Gershwin M.E., Ma X. (2017). Bile acids and intestinal microbiota in autoimmune cholestatic liver diseases. Autoimmun. Rev..

[79] Han S., Zhang R., Jain R., Shi H., Zhang L., Zhou G., Sangwung P., Tugal D., Atkins G.B., Prosdocimo D.A. (2015). Circadian control of bile acid synthesis by a KLF15-Fgf15 axis. Nat. Commun..

[80] Denson L.A., Sturm E., Echevarria W., Zimmerman T.L., Makishima M., Mangelsdorf D.J., Karpen S.J. (2001). The orphan nuclear receptor, shp, mediates bile acid-induced inhibition of the rat bile acid transporter, ntcp. Gastroenterology.

[81] Out C., Patankar J.V., Doktorova M., Boesjes M., Bos T., de Boer S., Havinga R., Wolters H., Boverhof R., van Dijk T.H. (2015). Gut microbiota inhibit Asbt-dependent intestinal bile acid reabsorption via Gata4. J. Hepatol..

[82] Beuling E., Kerkhof I.M., Nicksa G.A., Giuffrida M.J., Haywood J., aan de Kerk D.J., Piaseckyj C.M., Pu W.T., Buchmiller T.L., Dawson P.A. (2010). Conditional Gata4 deletion in mice induces bile acid absorption in the proximal small intestine. Gut.

[83] Quinn R.A., Melnik A.V., Vrbanac A., Fu T., Patras K.A., Christy M.P., Bodai Z., Belda-Ferre P., Tripathi A., Chung L.K. (2020). Global chemical effects of the microbiome include new bile-acid conjugations. Nature.

[84] Sannasiddappa T.H., Lund P.A., Clarke S.R. (2017). In Vitro Antibacterial Activity of Unconjugated and Conjugated Bile Salts on Staphylococcus aureus. Front. Microbiol..

[85] Sung J.Y., Shaffer E.A., Costerton J.W. (1993). Antibacterial activity of bile salts against common biliary pathogens. Effects of hydrophobicity of the molecule and in the presence of phospholipids. Dig. Dis. Sci..

[86] Coleman R., Lowe P.J., Billington D. (1980). Membrane lipid composition and susceptibility to bile salt damage. Biochim. Biophys. Acta.

[87] Inagaki T., Moschetta A., Lee Y.K., Peng L., Zhao G., Downes M., Yu R.T., Shelton J.M., Richardson J.A., Repa J.J. (2006). Regulation of antibacterial defense in the small intestine by the nuclear bile acid receptor. Proc. Natl. Acad. Sci. USA.

[88] Yang M., Gu Y., Li L., Liu T., Song X., Sun Y., Cao X., Wang B., Jiang K., Cao H. (2021). Bile Acid-Gut Microbiota Axis in Inflammatory Bowel Disease: From Bench to Bedside. Nutrients.

[89] Li T., Chiang J.Y. (2014). Bile acid signaling in metabolic disease and drug therapy. Pharmacol. Rev..

[90] Kurdi P., Kawanishi K., Mizutani K., Yokota A. (2006). Mechanism of growth inhibition by free bile acids in lactobacilli and bifidobacteria. J. Bacteriol..

[91] Yokota A., Fukiya S., Islam K.B., Ooka T., Ogura Y., Hayashi T., Hagio M., Ishizuka S. (2012). Is bile acid a determinant of the gut microbiota on a high-fat diet?. Gut Microbes.

[92] Xu M., Cen M., Shen Y., Zhu Y., Cheng F., Tang L., Hu W., Dai N. (2021). Deoxycholic Acid-Induced Gut Dysbiosis Disrupts Bile Acid Enterohepatic Circulation and Promotes Intestinal Inflammation. Dig. Dis. Sci..

[93] Barrasa J.I., Olmo N., Lizarbe M.A., Turnay J. (2013). Bile acids in the colon, from healthy to cytotoxic molecules. Toxicol. Vitro.

[94] Ovadia C., Perdones-Montero A., Fan H.M., Mullish B.H., McDonald J.A.K., Papacleovoulou G., Wahlstrom A., Stahlman M., Tsakmaki A., Clarke L.C.D. (2020). Ursodeoxycholic acid enriches intestinal bile salt hydrolase-expressing Bacteroidetes in cholestatic pregnancy. Sci. Rep..

[95] Aguirre A.M., Yalcinkaya N., Wu Q., Swennes A., Tessier M.E., Roberts P., Miyajima F., Savidge T., Sorg J.A. (2021). Bile acid-independent protection against *Clostridioides difficile* infection. PLoS Pathog..

[96] Gruner N., Mattner J. (2021). Bile Acids and Microbiota: Multifaceted and Versatile Regulators of the Liver-Gut Axis. Int. J. Mol. Sci..

[97] Islam K.B., Fukiya S., Hagio M., Fujii N., Ishizuka S., Ooka T., Ogura Y., Hayashi T., Yokota A. (2011). Bile acid is a host factor that regulates the composition of the cecal microbiota in rats. Gastroenterology.

[98] Ward J.B.J., Lajczak N.K., Kelly O.B., O’Dwyer A.M., Giddam A.K., Ni Gabhann J., Franco P., Tambuwala M.M., Jefferies C.A., Keely S. (2017). Ursodeoxycholic acid and lithocholic acid exert anti-inflammatory actions in the colon. Am. J. Physiol. Gastrointest. Liver Physiol..

[99] Ghouri Y.A., Tahan V., Shen B. (2020). Secondary causes of inflammatory bowel diseases. World J. Gastroenterol..

[100] Matsuoka K., Kanai T. (2015). The gut microbiota and inflammatory bowel disease. Semin. Immunopathol..

[101] Imhann F., Vich Vila A., Bonder M.J., Fu J., Gevers D., Visschedijk M.C., Spekhorst L.M., Alberts R., Franke L., van Dullemen H.M. (2018). Interplay of host genetics and gut microbiota underlying the onset and clinical presentation of inflammatory bowel disease. Gut.

[102] Lee T., Clavel T., Smirnov K., Schmidt A., Lagkouvardos I., Walker A., Lucio M., Michalke B., Schmitt-Kopplin P., Fedorak R. (2017). Oral versus intravenous iron replacement therapy distinctly alters the gut microbiota and metabolome in patients with IBD. Gut.

[103] Sinha S.R., Haileselassie Y., Nguyen L.P., Tropini C., Wang M., Becker L.S., Sim D., Jarr K., Spear E.T., Singh G. (2020). Dysbiosis-Induced Secondary Bile Acid Deficiency Promotes Intestinal Inflammation. Cell Host Microbe.

[104] Duboc H., Rajca S., Rainteau D., Benarous D., Maubert M.A., Quervain E., Thomas G., Barbu V., Humbert L., Despras G. (2013). Connecting dysbiosis, bile-acid dysmetabolism and gut inflammation in inflammatory bowel diseases. Gut.

[105] Nishida A., Nishino K., Sakai K., Owaki Y., Noda Y., Imaeda H. (2021). Can control of gut microbiota be a future therapeutic option for inflammatory bowel disease?. World J. Gastroenterol..

[106] Zuo T., Ng S.C. (2018). The Gut Microbiota in the Pathogenesis and Therapeutics of Inflammatory Bowel Disease. Front. Microbiol..

[107] Xu M., Shen Y., Cen M., Zhu Y., Cheng F., Tang L., Zheng X., Kim J.J., Dai N., Hu W. (2021). Modulation of the Gut Microbiota-farnesoid X Receptor Axis Improves Deoxycholic Acid-induced Intestinal Inflammation in Mice. J. Crohns Colitis.

[108] Van den Bossche L., Hindryckx P., Devisscher L., Devriese S., Van Welden S., Holvoet T., Vilchez-Vargas R., Vital M., Pieper D.H., Vanden Bussche J. (2017). Ursodeoxycholic Acid and Its Taurine- or Glycine-Conjugated Species Reduce Colitogenic Dysbiosis and Equally Suppress Experimental Colitis in Mice. Appl. Environ. Microbiol..

[109] Sung H., Ferlay J., Siegel R.L., Laversanne M., Soerjomataram I., Jemal A., Bray F. (2021). Global Cancer Statistics 2020: GLOBOCAN Estimates of Incidence and Mortality Worldwide for 36 Cancers in 185 Countries. CA Cancer J. Clin..

[110] Arnold C.N., Goel A., Blum H.E., Boland C.R. (2005). Molecular pathogenesis of colorectal cancer: Implications for molecular diagnosis. Cancer.

[111] Wang T., Cai G., Qiu Y., Fei N., Zhang M., Pang X., Jia W., Cai S., Zhao L. (2012). Structural segregation of gut microbiota between colorectal cancer patients and healthy volunteers. ISME J..

[112] Cao H., Xu M., Dong W., Deng B., Wang S., Zhang Y., Wang S., Luo S., Wang W., Qi Y. (2017). Secondary bile acid-induced dysbiosis promotes intestinal carcinogenesis. Int. J. Cancer.

[113] Pai R., Tarnawski A.S., Tran T. (2004). Deoxycholic acid activates beta-catenin signaling pathway and increases colon cell cancer growth and invasiveness. Mol. Biol. Cell.

[114] Cheng K., Raufman J.P. (2005). Bile acid-induced proliferation of a human colon cancer cell line is mediated by transactivation of epidermal growth factor receptors. Biochem. Pharmacol..

[115] Chen D., Jin D., Huang S., Wu J., Xu M., Liu T., Dong W., Liu X., Wang S., Zhong W. (2020). Clostridium butyricum, a butyrate-producing probiotic, inhibits intestinal tumor development through modulating Wnt signaling and gut microbiota. Cancer Lett..

[116] Chang C.W., Lee H.C., Li L.H., Chiang Chiau J.S., Wang T.E., Chuang W.H., Chen M.J., Wang H.Y., Shih S.C., Liu C.Y. (2020). Fecal Microbiota Transplantation Prevents Intestinal Injury, Upregulation of Toll-Like Receptors, and 5-Fluorouracil/Oxaliplatin-Induced Toxicity in Colorectal Cancer. Int. J. Mol. Sci..

[117] Wang Y., Li H. (2022). Gut microbiota modulation: A tool for the management of colorectal cancer. J. Transl. Med..

[118] Garrido A., Djouder N. (2021). Cirrhosis: A Questioned Risk Factor for Hepatocellular Carcinoma. Trends Cancer.

[119] Yu L.X., Schwabe R.F. (2017). The gut microbiome and liver cancer: Mechanisms and clinical translation. Nat. Rev. Gastroenterol. Hepatol..

[120] Dapito D.H., Mencin A., Gwak G.Y., Pradere J.P., Jang M.K., Mederacke I., Caviglia J.M., Khiabanian H., Adeyemi A., Bataller R. (2012). Promotion of hepatocellular carcinoma by the intestinal microbiota and TLR4. Cancer Cell.

[121] Lapidot Y., Amir A., Nosenko R., Uzan-Yulzari A., Veitsman E., Cohen-Ezra O., Davidov Y., Weiss P., Bradichevski T., Segev S. (2020). Alterations in the Gut Microbiome in the Progression of Cirrhosis to Hepatocellular Carcinoma. mSystems.

[122] Xie G., Wang X., Huang F., Zhao A., Chen W., Yan J., Zhang Y., Lei S., Ge K., Zheng X. (2016). Dysregulated hepatic bile acids collaboratively promote liver carcinogenesis. Int. J. Cancer.

[123] Schwabe R.F., Greten T.F. (2020). Gut microbiome in HCC—Mechanisms, diagnosis and therapy. J. Hepatol..

[124] Zhang H.L., Yu L.X., Yang W., Tang L., Lin Y., Wu H., Zhai B., Tan Y.X., Shan L., Liu Q. (2012). Profound impact of gut homeostasis on chemically-induced pro-tumorigenic inflammation and hepatocarcinogenesis in rats. J. Hepatol..

[125] Li J., Sung C.Y., Lee N., Ni Y., Pihlajamaki J., Panagiotou G., El-Nezami H. (2016). Probiotics modulated gut microbiota suppresses hepatocellular carcinoma growth in mice. Proc. Natl. Acad. Sci. USA.

[126] Dai H., Otsuka A., Tanabe K., Yanagita T., Nakayama J., Kitagaki H. (2022). Glucosylceramide Changes Bacterial Metabolism and Increases Gram-Positive Bacteria through Tolerance to Secondary Bile Acids In Vitro. Int. J. Mol. Sci..

[127] D’Haens G.R., Jobin C. (2019). Fecal Microbial Transplantation for Diseases Beyond Recurrent Clostridium Difficile Infection. Gastroenterology.

[128] Zhang L., Chu J., Hao W., Zhang J., Li H., Yang C., Yang J., Chen X., Wang H. (2021). Gut Microbiota and Type 2 Diabetes Mellitus: Association, Mechanism, and Translational Applications. Mediat. Inflamm..

[129] Larsen N., Vogensen F.K., van den Berg F.W., Nielsen D.S., Andreasen A.S., Pedersen B.K., Al-Soud W.A., Sorensen S.J., Hansen L.H., Jakobsen M. (2010). Gut microbiota in human adults with type 2 diabetes differs from non-diabetic adults. PLoS ONE.

[130] Mantovani A., Dalbeni A., Peserico D., Cattazzo F., Bevilacqua M., Salvagno G.L., Lippi G., Targher G., Danese E., Fava C. (2021). Plasma Bile Acid Profile in Patients with and without Type 2 Diabetes. Metabolites.

[131] Huang H.H., Lee W.J., Chen S.C., Chen T.F., Lee S.D., Chen C.Y. (2019). Bile Acid and Fibroblast Growth Factor 19 Regulation in Obese Diabetics, and Non-Alcoholic Fatty Liver Disease after Sleeve Gastrectomy. J. Clin. Med..

[132] Han J.L., Lin H.L. (2014). Intestinal microbiota and type 2 diabetes: From mechanism insights to therapeutic perspective. World J. Gastroenterol..

[133] Sansome D.J., Xie C., Veedfald S., Horowitz M., Rayner C.K., Wu T. (2020). Mechanism of glucose-lowering by metformin in type 2 diabetes: Role of bile acids. Diabetes Obes. Metab..

[134] Forslund K., Hildebrand F., Nielsen T., Falony G., Le Chatelier E., Sunagawa S., Prifti E., Vieira-Silva S., Gudmundsdottir V., Pedersen H.K. (2015). Disentangling type 2 diabetes and metformin treatment signatures in the human gut microbiota. Nature.

[135] Huda M.N., Kim M., Bennett B.J. (2021). Modulating the Microbiota as a Therapeutic Intervention for Type 2 Diabetes. Front. Endocrinol..

[136] McCartney C.R., Marshall J.C. (2016). Clinical Practice. Polycystic Ovary Syndrome. N. Engl. J. Med..

[137] Macut D., Bjekic-Macut J., Rahelic D., Doknic M. (2017). Insulin and the polycystic ovary syndrome. Diabetes Res. Clin. Pract..

[138] Liu R., Zhang C., Shi Y., Zhang F., Li L., Wang X., Ling Y., Fu H., Dong W., Shen J. (2017). Dysbiosis of Gut Microbiota Associated with Clinical Parameters in Polycystic Ovary Syndrome. Front. Microbiol..

[139] Guo J., Shao J., Yang Y., Niu X., Liao J., Zhao Q., Wang D., Li S., Hu J. (2022). Gut Microbiota in Patients with Polycystic Ovary Syndrome: A Systematic Review. Reprod. Sci..

[140] Zhou L., Ni Z., Yu J., Cheng W., Cai Z., Yu C. (2020). Correlation Between Fecal Metabolomics and Gut Microbiota in Obesity and Polycystic Ovary Syndrome. Front. Endocrinol..

[141] Yang X., Wu R., Qi D., Fu L., Song T., Wang Y., Bian Y., Shi Y. (2021). Profile of Bile Acid Metabolomics in the Follicular Fluid of PCOS Patients. Metabolites.

[142] Zhang B., Shen S., Gu T., Hong T., Liu J., Sun J., Wang H., Bi Y., Zhu D. (2019). Increased circulating conjugated primary bile acids are associated with hyperandrogenism in women with polycystic ovary syndrome. J. Steroid Biochem. Mol. Biol..

[143] Qi X., Yun C., Sun L., Xia J., Wu Q., Wang Y., Wang L., Zhang Y., Liang X., Wang L. (2019). Gut microbiota-bile acid-interleukin-22 axis orchestrates polycystic ovary syndrome. Nat. Med..

[144] He F.F., Li Y.M. (2020). Role of gut microbiota in the development of insulin resistance and the mechanism underlying polycystic ovary syndrome: A review. J. Ovarian Res..

[145] Zhang F., Ma T., Cui P., Tamadon A., He S., Huo C., Yierfulati G., Xu X., Hu W., Li X. (2019). Diversity of the Gut Microbiota in Dihydrotestosterone-Induced PCOS Rats and the Pharmacologic Effects of Diane-35, Probiotics, and Berberine. Front. Microbiol..

[146] Guo Y., Qi Y., Yang X., Zhao L., Wen S., Liu Y., Tang L. (2016). Association between Polycystic Ovary Syndrome and Gut Microbiota. PLoS ONE.

